# Supplementary material for: Rising Incidence of Non-Cardia Gastric Cancer among Young Women in the United States, 2000–2018: A Time-Trend Analysis Using the USCS Database
Source: Cancers (Basel). 2023 Apr 13;15(8):2283. doi: 10.3390/cancers15082283 (PMC10137096; doi:10.3390/cancers15082283)
Supplement: Supplementary file 1 [file cancers-15-02283-s001.zip › cancers-2325140-supplementary.pdf]

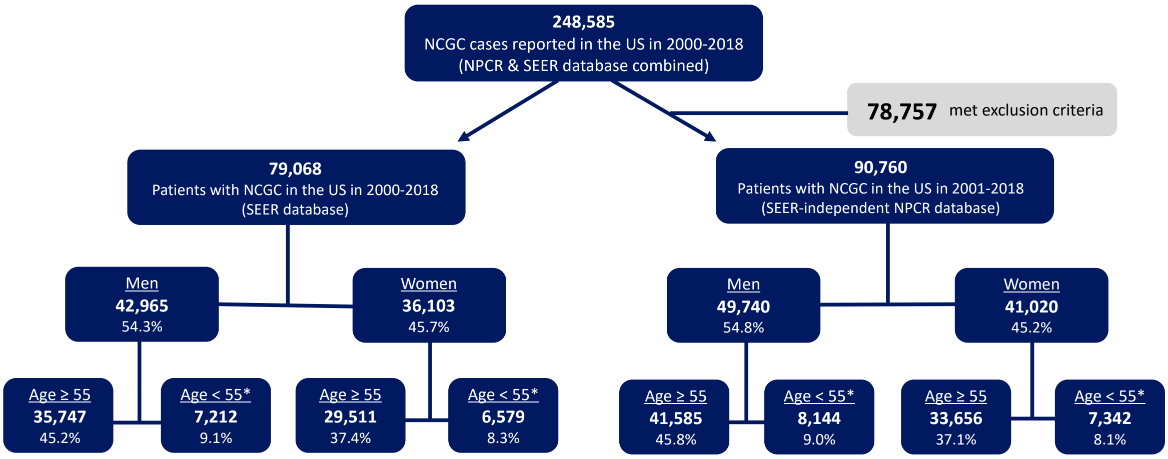

Supplementary Figure S1. Study sample inclusion and distribution by database, sex, and age.
- Data are presented as n, % of total sample per database.
- Exclusion criteria: cancer registries from NPCR database that also reported data in part or full to SEER (Alaska, California, Connecticut, Georgia, Hawaii, Idaho, Iowa, Kentucky, Louisiana, Massachusetts, New Jersey, New Mexico, New York, Utah, and Washington)
*Age < 55 groups exclude patients of ages 0-14 years

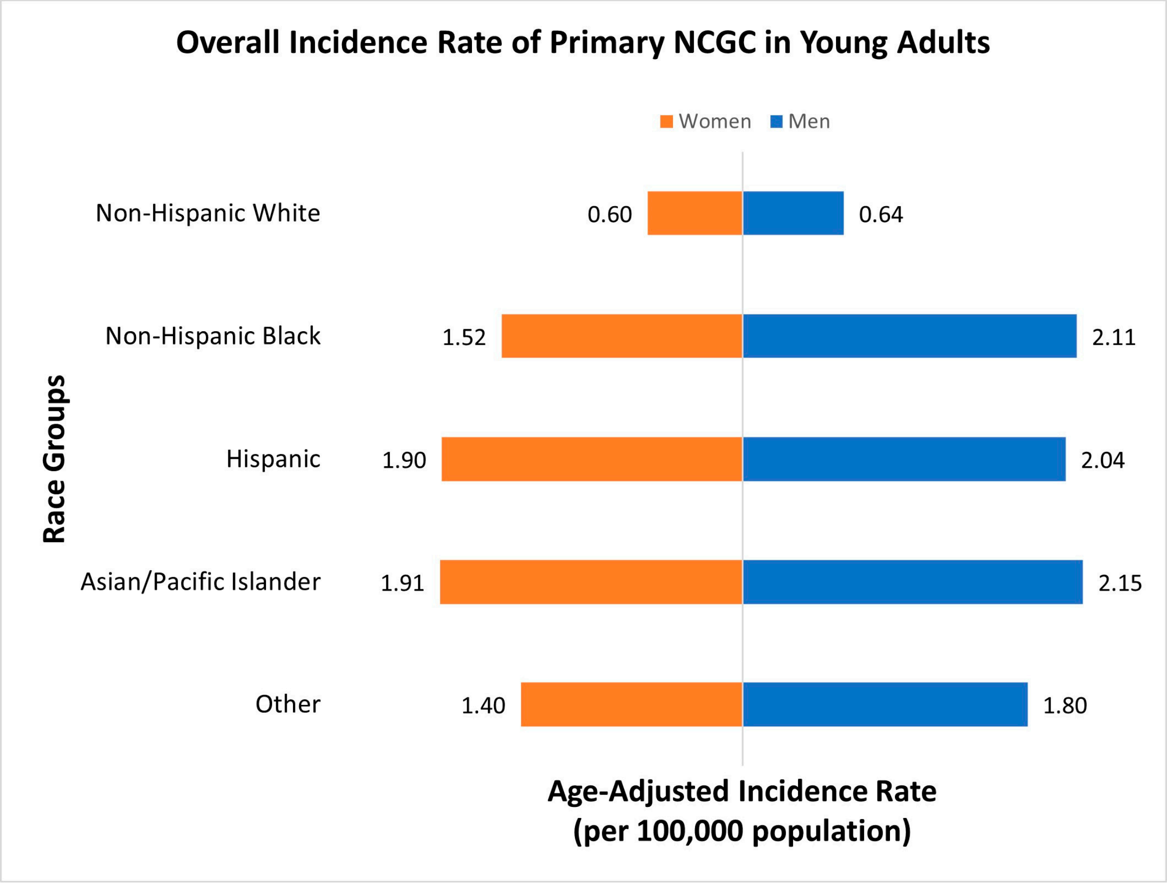

Supplementary Figure S2. Overall incidence rates of primary NCGC in young adults (<55 years old) by race groups from SEER database (2000–2018).

- Data are presented with delay-adjusted incidence rate (per 100,000 population).
